# *f* = m*a: A Framework for Investigating the Regulation of Replication Timing

**DOI:** 10.3390/genes13020249

**Published:** 2022-01-28

**Authors:** Nicholas Rhind

**Affiliations:** Biochemistry and Molecular Biology, University of Massachusetts Medical School, Worcester, MA 01605, USA; nick.rhind@umassmed.edu

**Keywords:** replication timing, MCM, origin firing, stochastic model

## Abstract

Stochastic models of replication timing posit that origin firing timing is regulated by origin firing probability, with early-firing origins having a high probability of firing and late-firing origins having a lower probability. However, they offer no insight into why one origin should have a higher firing probability than another. Here, a simple framework is suggested for how to approach the question by noting that the firing probability (*f*) must be the product of the stoichiometry of the MCM replicative helicase loaded at the origin (*m*) and the probability with which that MCM is activated (*a*). This framework emphasizes that mechanistic understanding of replication timing must focus on MCM loading and activation and can be simplified to the equation *f* = m*a.

## 1. Introduction

Simplification is the heart of science. In some cases, the simplification is a rigorous approximation, such as the relationship between force, mass, and acceleration in Newton’s Second Law of Motion: *F* = m*a. In other cases, the simplifications are conceptual ones that allow a complicated problem to be broken into simpler, more tractable problems. This perspective proposes a simplified framework for thinking about the complicated problem of how replication origin timing is regulated.

The timing of origin firing regulates genome-wide replication kinetics in eukaryotes, with origins in some parts of the genome firing early and in others firing later [1]. However, origin firing is also heterogeneous. Any given origin fires only in a fraction of S phases and there is a broad distribution of firing times for all origins, with early origins occasionally firing late and late origins occasionally firing early [2,3,4,5,6,7,8]. These apparently conflicting observations can be reconciled by a stochastic model in which the defining parameter for origin firing is not timing but rather probability [9,10,11]. In such a model, an early origin has a higher probability of firing and thus, on average, fires earlier, whereas a late/inefficient origin has a low probability of firing and so fires, on average, late. There is strong evidence for such a model in yeast [2,3,5,7] and increasing evidence for it in vertebrate cells [6,8,12,13,14,15,16].

Although a stochastic model can describe the experimental observations of reproducible replication timing and heterogeneous origin firing, it provides no insight into the key mechanistic question it raises: How is the probability of origin firing regulated? One way to approach the problem is to observe that the rate-limiting step for origin firing is the activation of the MCM replicative helicase. The biochemistry of MCM activation and its incorporation into the processive CMG (Cdc45-MCM-GINS) helicase complex is well understood [17]. Those biochemical details notwithstanding, the important conclusion is that the probability of origin activation depends on, and only on, the probability that an MCM complex is loaded at an origin and the probability that the MCM complex is activated during S phase. Although this conclusion may seem self-evident, it is a powerful way to focus mechanistic studies of origin timing regulation. If a regulatory pathway can be shown to affect the loading of MCM, but not its activation probability, or vice versa, that knowledge significantly constrains the mechanisms by which that pathway can work. In particular, if one wants to understand the mechanism by which developmental regulation or cellular perturbation affects origin timing, one needs to understand both whether it affects MCM loading or activation and how it does so. Thus, it is useful to focus on the formula *f* = m*a.

## 2. *f*: The Firing Probability of an Origin

The heterogeneous nature of origin firing means that any one origin will fire only in a fraction of cells. That fraction can be as high as 90% for some efficient budding yeast origins [18], but is typically closer to 5 or 10% in mammalian cells [4,6]. The reason that not all origins fire early in S phase is that their activation is controlled by rate-limiting activators, which are required to transform an inactive MCM loaded at an origin into an active CMG replicative helicase [19,20]. The regulation of origin firing by limiting factors explains why origins fire with low probability. The observed diffusibility of limiting factors [21,22,23], can explain why origin firing is stochastic. The limiting factors diffuse randomly throughout the nucleus and when they interact productively with an MCM loaded at an origin, that origin fires. In budding yeast, the limiting activators are primarily the Sld2 and Sld3 initiation factors, their binding partner, Dpb11, and Dbf4, the regulatory subunit of DDK, the Dbf4-dependent kinase [19,20]. In fission yeast, they are a CMG subunit, Cdc45, and DDK [22,24]. However, the exact identity of the limiting activators is not important. Stochastic models are compatible with any limiting activator, as long as it is freely diffusible and can activate multiple sequential origins [11].

The varying probability of origin firing, which is observed in yeast and humans [2,5,6], provides a mechanistic explanation for the regulation of replication timing, as described above, both for the well-defined origins of budding yeast and the broad initiation zones of mammalian cells [3,7,11,13]. In particular, in stochastic models, the probability of origin firing is the key regulated parameter that controls replication timing. (For the purpose of this perspective, the firing probability of an origin is abbreviated *f*. In the replication-kinetics modeling literature, this parameter is referred to as the initiation probability, *I*. In that literature, *f* is used instead to represent the fraction of the genome replicated at any point in time [25].) In this context, it is important to distinguish origin firing probability (the probability of an origin firing over a given time interval) from origin efficiency (the fraction of cells in which an origin fires). The two are distinct because origins are often passively replicated. For instance, a high-probability origin near another high-probability origin will often be passively replicated by its neighbors and thus may have a low firing efficiency. The distinction is not so important for general conceptual discussions, but it is crucial for making realistic detailed models.

## 3. *m*: The MCM Loading Stoichiometry

Loading of the MCM replicative helicase is the necessary and sufficient biochemical step that establishes a potential replication origin [26]. MCM is a heterohexameric cylinder that is loaded around double-stranded DNA in an inactive form during G1 by the origin recognition complex (ORC) [27]. Two MCM hexamers are loaded sequentially at origins to form a double hexamer [28], providing one MCM for each of the two replication forks initiated at each origin. Therefore, loading of a single MCM double-hexamer complex suffices to establish an origin. Moreover, once MCM is loaded at an origin, it remains stably associated [29], providing durable origin potential to the locus. However, MCM loading is not a binary characteristic: A locus does not either always have an MCM complex loaded in G1 or never have an MCM complex loaded in G1. Instead, MCM stoichiometry at origins can vary. Clearly, if an origin has an MCM loaded in only half of the cells in a population, then that origin can fire in, at most, half of the cells. More generally, the fraction of cells in which MCM is loaded at an origin is a key regulator of origin firing probability. It has been noted that varying the fraction of cells in which an origin has an MCM complex loaded, a parameter referred to as origin competence, can explain the variable firing probabilities of euchromatic budding yeast origins and thus explain their replication timing [3]. Alternatively, it has been proposed that more than one MCM double hexamer may be loaded at these origins [7]. In the latter case, instead of varying from zero to one, MCM stoichiometry would vary from zero to many. Experimental data suggests that efficient budding yeast origins may average three MCM complexes [30], although others have argued that many budding yeast origins have at most one MCM double hexamer loaded [12]. Regardless, whether MCM stoichiometry varies from zero to one or from zero to many does not matter for the purpose of this perspective. In either case, the higher the relative stoichiometry, the higher the probability of origin activation.

Little is known about how MCM stoichiometry may be regulated. Regulation of ORC activity is certainly a leading candidate. In yeast, ORC occupancy correlates with replication timing, although not strongly [31,32,33], suggesting that ORC occupancy is part of the answer. ORC occupancy could be controlled by its affinity for its binding site or interaction with other *cis*-acting factors [33,34,35]. It is also possible that ORC-specific activity could be modulated by *cis*-acting regulators. Alternatively, chromatin structure could have both permissive and instructive roles in regulating MCM stoichiometry [31,36,37]. Future work on the regulation of MCM stoichiometry will provide important insights into the regulation of replication kinetics.

## 4. *a*: The Activatability of the MCM

The rate-limiting step for origin activation during S phase is the activation of a loaded MCM double hexamer. If every MCM were activated during S phase, then the probability of origin firing would simply be determined by MCM loading stoichiometry. However, not every MCM is activated during S phase. In fact, in budding yeast, it is estimated that only 10% of MCMs are activated. Moreover, it is clear that not all MCMs are activated with the same probability. For instance, in budding yeast, many MCMs are loaded at subtelomeric origins [30,38], but these origins fire late [39], suggesting that the MCM loaded there fire with low probability. This late firing is dependent on telomeric heterochromatin [40,41]. Likewise, in metazoan cells, replication timing is closely correlated with chromatin state, with heterochromatic regions replicating later than euchromatic regions [1]. Heterochromatin, in both yeast and metazoans, reduces the rate of MCM activation by Rif1-dependent recruitment of phosphatases, which counteracts the activating phosphorylations of the S-phase kinases [42,43,44,45,46,47]. Heterochromatin is often described as inhibiting activities such as transcription and replication initiation by physically restricting access of heterochromatic DNA to soluble activating proteins. However, the establishment of heterochromatin as a region of locally high phosphatase activity plausibly explains heterochromatin’s inhibitory effect on the initiation of both replication and transcription.

Beyond the repressive effect of heterochromatin, the probability of MCM activation can vary across euchromatic origins and can be positively regulated. In budding yeast, Fkh1 (Forkhead1) has been proposed to facilitate the early firing of individual origins by recruiting initiation factors [48]. Likewise, in both budding and fission yeast, centromeric origins fire early because they recruit DDK [49,50], and direct tethering of DDK near a fission yeast origin increases its probability of firing [22]. There is every reason to believe that these examples are just the beginning and that there are many other mechanisms for both the positive and negative regulation of MCM activation to advance or delay replication timing.

## 5. Application of the Formula

Replication timing has been proposed to regulate critical aspects of nuclear metabolism, such as gene expression, chromatin structure, and genome evolution [51,52,53]. However, how timing is regulated is still an open question [1]. Dividing the general question about replication timing into specific questions about the regulation of MCM loading and MCM activation makes the question experimentally more tractable. For instance, in situations in which *trans*-acting factors [32,48] or *cis*-acting sequences [54] have been shown to affect replication timing, subsequent investigation should be focused on whether and how MCM loading or activation is affected. It also makes it clear that in different parts of different genomes, different mechanisms may be at work. For instance, in mammalian genomes, in which timing correlates well with chromatin structure, MCM activatability may play the dominant role in regulating replication timing, although the rate of MCM loading has been reported to vary between euchromatin and heterochromatin [55]. In yeast genomes, which are primarily euchromatic—or, for that matter, in euchromatic regions of mammalian genomes—MCM activation may be more uniform and MCM loading may play a larger role in replicating timing [12,30,56]. Exceptions from these general trends may provide specific examples of timing regulation mechanisms. In any case, focusing on the specific questions of how MCM loading is regulated and what affects the probability of activation of loaded MCM would transform studies that describe the phenomenology of replication time into studies that contribute to our understanding of the mechanisms that regulate replication timing. Therefore, it is useful to think of the problem of replication timing as framed by *f* = m*a.

## Data Availability

Data sharing not applicable.
